# On the Use of Cold Water in Scarlatina Maligna

**Published:** 1849-11

**Authors:** 


*On the Use of Cold Water in Scarlatina Maligna.* By Wm. H. Anderson, M. D., of Mobile, Alabama.

In no part of the United States, or of Europe, have we seen scarlet fever so successfully treated as it is in Holland, and the cause of the success of the Dutch physicians is, in our opinion, the fact that they pursue the most rational mode of treatment. They may have not investigated the disease so philosophically or so learnedly as their neighbors, but they certainly have fallen upon a more reliable treatment, especially in scarlatina maligna, than any of the nations that surround them.

Scarlet fever we believe to be caused by a peculiar virus which floats in the atmosphere, and which enters the human system by some unknown means, preferring infant to adult life, acting, as a general rule, with more severity on the robust and plethoric, than on the weakly and anæmic, giving rise to a most malignant disease in a few, while the attendant symptoms are light and unimportant on the large majority, sometimes easily eradicated from the system by the efforts of nature and the intervention of art; at other times bidding defiance to the strongest constitutions, and unaffected by the most powerful remedial measures. Whether this virus be of animal or of vegetable origin, we leave to the advocates of the animalcule and the cryptogamic schools to discuss. In fact, they have largely discussed it, and are still as much in the dark as when they commenced. Their investigations, however, are commendable, and, if patiently pursued, will possibly lead to happy results. The virus having entered the economy, spends its action principally, perhaps exclusively, on the blood, and when it gives rise to malignant disease, the blood is profoundly altered; the salts are diminished; the globules decreased, and the fibrin, at first augmented, is, towards the close, rapidly lessened and materially changed. The alteration of the solids is owing to the circulation of vitiated fluids. As long as the virus remains unchanged in the system, these effects will be produced, and the important question to be asked is, can we expel the poison or lessen its virulence? Experience has proved that in many instances, we cannot expel it—in some, the duration is so short that there is no time for remedies to act. In such cases, scarlatina resembles charbon and malignant cholera. In other cases, the virus hangs with such tenacity in the system, that the organs of excretion although assisted by their appropriate stimuli, labor in vain to throw it off. Are these forms of the disease to be abandoned as hopeless? With regard to the first, viz: that in which the disease rapidly runs its course, we answer, certainly not. There is a remedy simple, yet powerful in its nature, which can often either modify the virulence of the poison, or enable the blood to resist the changes produced by it. This agent is *cold water* poured over the body, or, in other words, what is known in medicine as the *cold douche.* When and where the application of cold water in scarlatina maligna originated, we are unable to say. The first authentic account, that the writer has been able to find, i that given by Erinboker, a Dutch physician, who during the middle of th last century, used the cold douche with great success in a large number cases. From time to time, it has been successively tried in various cou tries, and extravagant eulogiums upon the practice have been writte Many well attested facts and statistical tables have been given, which pro that it is a remedy of great value, and yet, so great is the aversion to use, merely because it looks like heroic practice, that it is often neglecte

In other forms of malignant fever, attended by nervous and cerebral excitement, and accompanied by alteration of the blood, the cold douche is used with the happiest results. Even the French, whose antipathy to any thing cold is only equalled by their ardent admiration of hot ptisanes and smoking poultices—even they will sometimes waive their national antipathy, and employ the cold douche in typhoid and other fevers accompanied by cerebral excitement, and acknowledge, too, the efficacy of the treatment, and yet we do not remember ever to have seen them use cold water in scarlatina maligna, although it was strongly indicated by experience, by analogy, and by circumstances. We are aware, however, that it has occasionally been used, both in Paris and in Lyons, with the most cheering success, but the cases have been too few to give currency to the treatment.

In England, many physicians have spoken favorably of the *cold douche*, and yet, from some unaccountable cause, the remedy has found its way into very few of the systematic treatises on practical medicine, or if mentioned at all, is glanced at in a very cursory manner. Some good monographs have been written on the subject, and the practice, though not much lauded by the standard writers, is often used by private practitioners. The article of Dr. Currie, as coming from an eminent physician, deserves attention. Dr. John Mason Good, in alluding to this article, says: "The great inquieiude that characterises this disease has induced many practitioners to try opium, but it rarely affords relief in any form or combination, and generally renders the head worse; acids, whether vegetable or mineral, are always grateful to the patient, and seem more than any other internal means to diminish the burning heat of the skin. But our chief dependence for this purpose must be upon Dr. Currie's bold and happy plan of employing *cold water* freely. Sponging will rarely be found sufficient, or rather will rarely be found of equal advantage with affusion; the fluid may, indeed, in this case, be repeated as fast as it returns. The refreshment is often instantaneous, and operates like a charm; and seems to show, as I had occasion to observe formerly, not merely a refrigerant, but an exhilarating power, as though the water was decomposed, and a part of its oxygen was swallowed greedily by the thirsty absorbents of the skin, which immediately becomes softer and more moist, as well as cooler."

We have been told by a pupil of the learned Frank, so long a distinguished lecturer in the University of Pavia, that toward the latter part of his life, he was in the habit of using diuretics and bark internally, and cold water externally, in the treatment of malignant scarlet fever. We do not find any allusion to this treatment in his celebrated work, "De morbis hom. cur. epit.," but this is owing to his having taken up the practice in the latter part of his life. Few practitioners enjoyed the same extensive practice that Frank did, and few, indeed, were gifted with the same philosophical mind, so that we regard his opinions as worthy of the highest consideration. His idea in using diuretics was to expel from the blood the morbific matter by the channel of the kidneys, while he used cold to calm the excitement, to act as a capillary astringent, and to enable the blood to resist the poisonous action of the virus. While we are no advocate of the doctrine of hydro-sudo-pathy, as indiscriminately practiced by its upholders in some parts of Germany, we must, as an impartial observer at two of their establishments, lend our testimony to their successful treatment of scarlet fever.

In the United States, cold affusion has been occasionally used in the disease in question, by respectable practitioners, and particularly by Dr. Bell, of Philadelphia, whose able article on the subject cannot be read with too much attention. The subject seems in this paper to be very philosophically handled, and the boldness with which the arguments are set forth, considering the novelty of the doctrine and the prejudices to be overcome, reflects great credit upon the author. From our conversation with Dr. Fiolini, of Milan, the pupil of *Frank*, we should infer that that distinguished practitioner entertained views very analogous to those of Dr. Bell. It is due to Dr. Chapman, of Philadelphia, to say, that he recommends cold affusion in the milder cases of Scarlatina, when there are great heat and excitement. Speaking of the severe *congestive stage*, he says: "It has occurred to me, extraordinary as it may seem, that even here, cold applications might possibly be beneficial. Considering the decisive control they exercise over the deleterious agency, productive of the disease, I was inclined to this conclusion, and the more confirmed in it, by the contemplation of some facts lending to it no insignificant support." Our own private practice in Scarlet Fever, has been too limited, for us to detail a sufficient number of cases to give value to our remarks, but we must not neglect to say, that we have several times used the cold douche, when the symptoms were quite alarming, and we had every reason to be pleased with the result. We know very well, that it is rather appalling to a mother, to see her child submitted to a remedy so *shocking* in its appearance, especially with the existing popular, though unphilosophical, notions of repelling eruptions; but we do not think, that a conscientious practitioner should suffer any undue timidity to prevent him from using a useful remedy, although it may appear bold, or even *rash* to those who are ignorant of his profession. The physician should be like the soldier, always cool and deliberate, and ever ready to be heroic when the hope is forlorn.

With regard to the modus operandi of the cold douche, we believe that its effects are manifold, but the ultimate action of the large majority of remedies is involved in so much mystery, that we will not, on the present occasion, do more than broach the subject. By what means cold water modifies the poison when it has entered the blood, or how it enables the blood to resist the changes which the virus occasions, is a subject too deep for us to discuss. The nearest approach that we can make to a plausible argument is, that we know that sudden changes of temperature have a marked effect on malaria, both animal and vegetable. Whether the impression of cold, when applied to the skin, has any destructive agency on the virus within the system, we do not pretend to say. But with regard to the physical effects of cold, as an astringent, we can speak with more certainty. We believe that applied externally, it is not only an astringent to the capillary system of the skin, but also, to the capillary system in general. That every internal viscus shares this astringent effect, we do not doubt. We have often seen the wildest delirium of meningitis calmed by its application. In what way could it have acted, other than by reducing the circulation? We have likewise seen the cold douche put a stop to uterine, pulmonary, and gastric hemorrhages of the most alarming character, and we do not know how it could do this, except by calming the circulation, and corrugating the capillaries that gave rise to the hemorrhage. When the hemorrhage was sthenic, we can account for its influence in another way, but when asthenic and passive, it must have acted on the capillaries themselves. With regard to the pharyngeal swelling that accompanies scarlatina, experience has proved to us, that it has been materially lessened by the cold douche, and we are decidedly of the opinion, that the same astringent and corrugating effect above alluded to, was the cause of the reduction. This latter, however, we believe to be of secondary importance. The calmative agency of the remedy, and its power to relieve the congestion of the brain and lungs, is of far greater value.

In making the *post mortem* examination of children, who have died within the first fifty or sixty hours of scarlatina maligna, we have found great congestion, and sometimes inflammation of the brain and its membranes. Indeed, in the majority of cases, the brain presented pretty much the appearance that it does in fatal cases of meningitis, and sufficient cause of death was found in the cranial cavity, without referring to the cervical or thoracic regions. In such cases, the hyperæmia of the cerebral organs is observed to come on quite early in the disease, and to be altogether antecedent to the thoracic congestion. There are substantial reasons for believing, that if this cerebral hyperœmia were controlled, the patient would, at any rate, be in a much better condition to withstand the attack. If it were a case of pure meningitis, cold would be used, but because there is swelling about the fauces, and an eruption upon the skin, it is thrown aside as hurtful and contra-indicated. Such reasoning, we think, is fallacious, and not in accordance with the true principles of an enlightened pathology.

With these remarks, we close the article. What we have said, has been set forth in a very cursory manner, and is intended more to call the attention of practitioners to the value of the remedy in question, than to enlighten them upon a subject with which they are doubtless well acquainted already. Having had our own attention turned particularly to the efficacy of the cold douche in scarlatina, we can confidently recommend it as one of the most powerful and effectual remedies which is known in the treatment of one of the most malignant and distressing maladies which it falls to the lot of the profession to treat.—*New Orleans Med. and Surg. Journal.*

